# Rare Splice Variants in Long Non-Coding RNAs

**DOI:** 10.3390/ncrna3030023

**Published:** 2017-07-05

**Authors:** Rituparno Sen, Gero Doose, Peter F. Stadler

**Affiliations:** 1 Bioinformatics Group, Department of Computer Science, and Interdisciplinary Center for Bioinformatics, University Leipzig, Härtelstrasse 16-18, D-04107 Leipzig, Germany; rituparno@bioinf.uni-leipzig.de; 2 ecSeq Bioinformatics, Brandvorwerkstraße 43, D-04275 Leipzig, Germany; gero.doose@ecseq.com; 3 German Centre for Integrative Biodiversity Research (iDiv) Halle-Jena-Leipzig, Competence Center for Scalable Data Services and Solutions, Leipzig Research Center for Civilization Diseases, and Leipzig Research Center for Civilization Diseases (LIFE), University Leipzig, Härtelstrasse 16-18, D-04107 Leipzig, Germany; 4 Max Planck Institute for Mathematics in the Sciences, Inselstraße 22, D-04103 Leipzig, Germany; 5 Fraunhofer Institute for Cell Therapy and Immunology, Perlickstrasse 1, D-04103 Leipzig, Germany; 6 Center for RNA in Technology and Health, University Copenhagen, Grønnegårdsvej 3, 1870 Frederiksberg C, Denmark; 7 Santa Fe Institute, 1399 Hyde Park Road, Santa Fe, NM 87501, USA

**Keywords:** lncRNA, splice junctions, GENCODE, lncRNA isoforms

## Abstract

Long non-coding RNAs (lncRNAs) form a substantial component of the transcriptome and are involved in a wide variety of regulatory mechanisms. Compared to protein-coding genes, they are often expressed at low levels and are restricted to a narrow range of cell types or developmental stages. As a consequence, the diversity of their isoforms is still far from being recorded and catalogued in its entirety, and the debate is ongoing about what fraction of non-coding RNAs truly conveys biological function rather than being “junk”. Here, using a collection of more than 100 transcriptomes from related B cell lymphoma, we show that lncRNA loci produce a very defined set of splice variants. While some of them are so rare that they become recognizable only in the superposition of dozens or hundreds of transcriptome datasets and not infrequently include introns or exons that have not been included in available genome annotation data, there is still a very limited number of processing products for any given locus. The combined depth of our sequencing data is large enough to effectively exhaust the isoform diversity: the overwhelming majority of splice junctions that are observed at all are represented by multiple junction-spanning reads. We conclude that the human transcriptome produces virtually no background of RNAs that are processed at effectively random positions, but is—under normal circumstances—confined to a well defined set of splice variants.

## 1. Introduction

Long non-coding RNAs (lncRNAs) are an important part of the mammalian transcriptome [1,2]. Although the set of lncRNAs that is well understood with respect to biological function and molecular mechanisms is still limited, it is rapidly expanding. Genes such as *ANRIL* [3,4], *HULC* [5], *MALAT1* [6], *TUG*1 [7], or *Xist* [8] may serve as examples. Widespread roles include—but are not limited to—interactions with chromatin to silence or activate chromatin [9,10], and the regulation of splicing [11]. Still, the coverage and precision of the lncRNA annotations lag behind the accurate maps of protein-coding genes. The GENCODE project [12] provides the most accurate transcript and gene annotation for the human genome. It is a combination of manual and automated annotation techniques which endeavours to list gene features from HAVANA and Ensembl datasets. Detailed surveys of expression patterns across many tissue and cell-types (e.g., [13,14,15]) provide evidence for intricate regulatory networks in which lncRNAs are key players.

There is mounting evidence, however, that lncRNA isoforms may differ drastically in their biological function [16,17]. Due to their usually low expression values, gene models for lncRNAs historically were often truncated—a situation that has only been improving recently. Notably, the recent lncRNA atlas by Hon et al. [15] is specifically aimed at providing accurate 5’ ends. The situation is still more difficult at the 3’ side, since long, large, unspliced 3’ regions make it difficult to determine complete transcript from Illumina data (e.g., [18,19]). Furthermore, lncRNAs such as *ANRIL* [16] exhibit complex patterns of alternative splicing. Even in extremely well-studied protein-coding loci, rare isoforms keep being discovered [20]. It thus remains an unresolved question to what extent the current maps of lncRNAs are complete, both in terms of the number of expressed transcripts per gene and in terms of the variability of their isoforms.

One component towards answering this question is to ask to what extent the transcript portfolio of a particular cell type has been mapped completely. Conversely, one asks in this context to what extent reported transcripts are noise. To address these issues, we investigate here a very large set of transcriptome data from B cell lymphomas. By virtue of aggregating hundreds of independently generated transcriptome datasets, we can study in detail if the set of detectable splice junctions converges to a consensus.

## 2. Results

A comparison of published annotation data showed substantial differences in the average number of exons per transcript, with some systematic trends over time. The recent Ensembl version 83 [21], which shares GENCODE v.24 [22] as basis of gene annotation, and reports slightly fewer introns per lncRNA gene than earlier versions. Earlier Ensembl versions report only a limited number of long intergenic non-coding RNA (lincRNA)  genes. Ensembl version 60 [23], for example, reports only 1443 lncRNAs, compared to 15,941 lncRNAs  in GENCODE v.24. The average number of introns is very close to the data reported for the much more complete GENCODE v.7 annotation. In contrast, the NONCODE database is very inclusive and provides more than an order of magnitude more entries. Correspondingly, the GENCODE annotation also exhibits a moderate decrease in the number of exons per lncRNA over time (Table 1). This is probably a consequence of the fact that more recently included lncRNA contain a larger fraction of intron-poor or even intronless RNAs.

To better understand the distribution and structure of rare isoforms in the human transcriptome, we systematically investigated the influence of the dataset size on the complexity of inferred gene structures. We focussed on RNA-Seq data from 111 lymphoma samples consisting of the different subtypes Burkitt lymphoma (BL), follicular lymphoma (FL), and diffuse large B cell lymphoma (DLBCL) that was published in the context of the ICGC MMML-Seq project [24]. The RNA-Seq data comprised of a total of more than 10 billion mapped reads. Since the RNA-seq data is not strand-specific, only non-overlapping lncRNA genes (lincRNAs) were considered for this analysis. Using GENCODE version 19 as reference, we obtained a set of 5257 lincRNAs that were detectably expressed in our lymphoma data.

**Figure 1:**
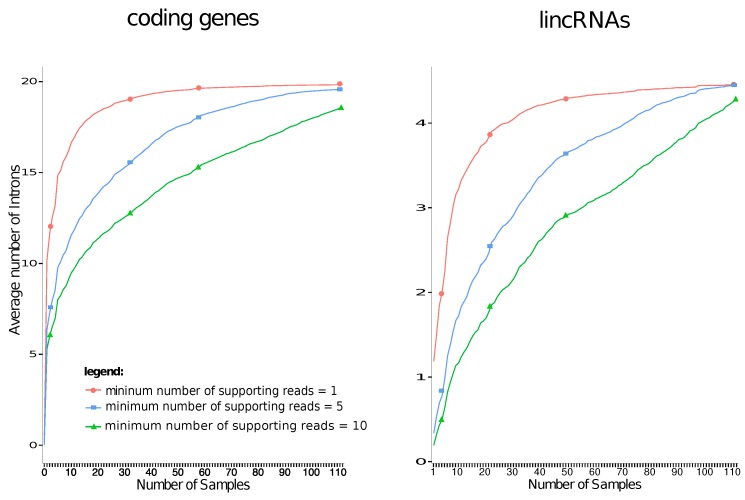
Saturation curves for the number introns as a function of the number of independent transcriptome samples. The lncRNAs data refer to the 1441 annotated genes in the lymphome dataset with at least one intron.

Figure 1 summarizes the effect of increasing coverage on the estimated average number of introns per gene locus. Here, only the subset of expressed lncRNAs with at least one annotated intron is used to reduce the chance of erroneously or ambiguously mapped reads. The data qualitatively reproduce the observation of the ENCODE project that there is a large difference in the average number of splice junctions between protein-coding loci and ncRNAs [25]. Although restricted to a quite narrowly defined cancer type, the average number of introns in lncRNAs is larger by about one than the GENCODE dataset, which uses a composite of a broad range of cell lines and tissues. While GENCODE v.19 reports an average of 3.21 introns for these genes, the analysis of the RNA-seq data reveals a mean of 4.29 introns. We can confirm that slightly more than 8% of the introns are novel. This is clearly an effect of the extreme sequencing depth of combined lymphome data. The curves also show that the data saturate very slowly, requiring dozens or even a hundred samples to reach the plateau value.

The data show that the detected splice junctions are unlikely to be noise, since the curves saturate well for all three minimum read support thresholds instead of showing linear growth, affirming their biological reality.

**Figure 2:**
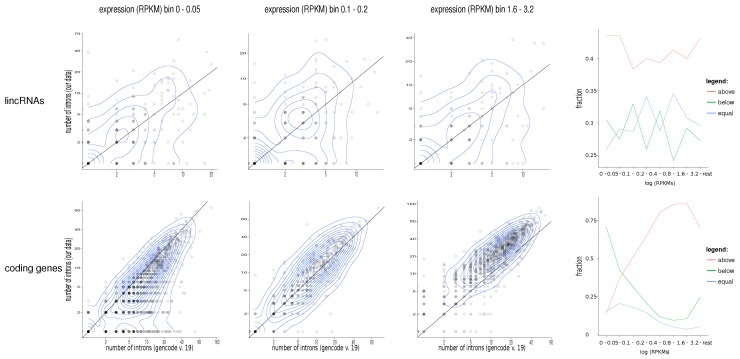
Scatterplots for different numbers of expression bins for long intergenic non-coding RNAs (lincRNAs) and coding genes. The diagonal, where x=y, is marked by a line. Points above the line are those genes for which we calculate more introns compared to GENCODE v.19. Only genes with at least one intron supported by at least 10 reads are considered here. The right-most column displays the fraction of genes that show more (red), the same (blue), or fewer (green) distinct splice junctions in the lymphoma data compared to GENCODE v.19. For the coding genes, there is a clear dependence of these fractions on the expression level: for highly expressed mRNAs, we systematically predict more (rare) splice variants. For mRNAs that are very lowly expressed in the lymphoma data set, GENCODE v.19 has more complex gene models. Overall, there are still more introns in our data set than annotated (Wilcoxon test $p<4\times 10^{-10}$). In contrast, we systematically see more introns in lincRNAs than annotated by GENCODE (Wilcoxon test $p<3\times 10^{-16}$), independent of the expression level. An alternative presentation of the r.h.s. panels showing data binned in 5-percentiles can be found in the Supplementary Material. RPKM: reads per kilobase and million reads.

In Figure 2, we compare the data in more detail with the GENCODE v.19 annotation, which was used here as the reference annotation dataset since the genes in the lymphome dataset have the largest overlap with this version. Alternative splicing is a very common phenomenon throughout the human genome [26,27], and expression levels of alternatively spliced isoforms are usually tissue-specific. It is not surprising that genes with multiple exons are more likely to have alternate splice sites [28,29]. For the case of protein-coding loci, we tend to miss some splice junctions at those loci that are extremely lowly expressed in the lymphome transcriptomes. This is not surprising, as rare variants are of course easier to detect in transcriptomes where they are more highly expressed; after all, the GENCODE annotation is a composite of vastly diverse cell types and tissues. It is interesting to note, however, that we systematically observe more introns at moderate reads per kilobase and million reads (RPKM) values, even from the very narrowly-defined cell types used here. This attests that large numbers of well-defined but rare isoforms have so far eluded annotation.

Comparing our data to existing GENCODE annotations, we find that lncRNAs exhibit systematically larger numbers of exons and introns. However, the discrepancy is moderate and applicable—in particular to lncRNAs that already have a large number of exons annotated. Around 41% of genes are found to have a greater  number of introns. Fourteen percent of the genes have at least one intron more, and 19% are calculated to have at least two introns more.

**Figure 3:**
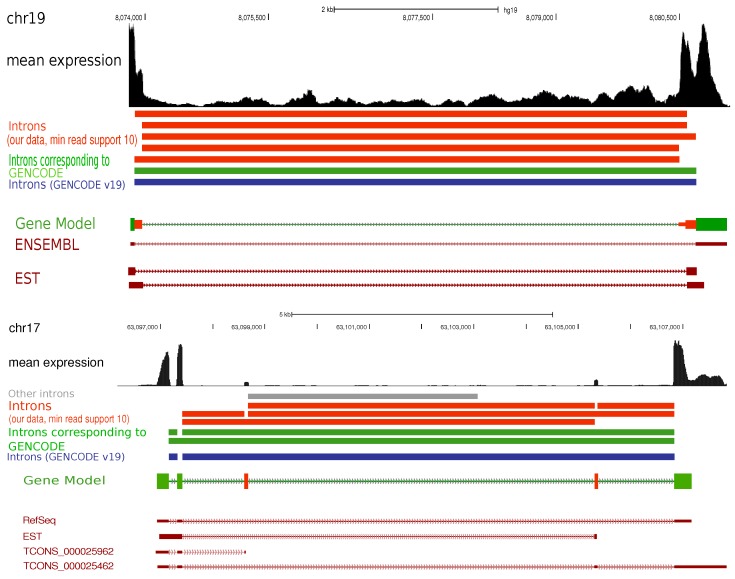
Two examples with previously unannotated splice junctions and introns. (**Top**) In ENSG00000267939, we find six introns and two additional exons compared to a single intron described in GENCODE v19. (**Below**) For ENSG00000263470 we find eight introns plus a likely false positive compared to two introns in GENCODE.

In Figure 3, we show two examples that appear to be substantially more complicated in our data than in the GENCODE annotation. No functional annotation is available at present for either locus. As the figure shows, at least some of the additional exons also appear in EST data tracks provided by the UCSC genome browser.

## 3. Concluding Remarks

We have shown that human transcriptome data harbour a large number of rare exons (and thus also introns) that have remained unannotated. Due to their low abundance, they appear only when data from large-scale experiments are pooled. As shown in Figure 1, they can nevertheless be reproduced very accurately. There is very little noise in these data, as shown by the near perfect saturation of the average number of splice junctions per gene. Transcriptional noise—whether biological or technical—would result in a linear increase of the number of detected junctions as a function of the size of the data set. If such a slope exists, it is too small to be detectable from our data, which comprise of more than $10^{10}$ reads. Therefore, we have to conclude that the current annotation of the human transcriptome is confined to a very well-defined set of splice variants. As a consequence, it is a meaningful and—in our view—worthwhile task to attempt the construction of an exhaustive atlas.

The well definedness of isoforms by itself does not imply that all isoforms carry biological functions. If the vast majority of isoforms are indeed non-functional junk, however, an explanation is needed for the precision of the processing and its restriction to very few splice sites.

## 4. Materials and Methods

The 111 RNA-seq lymphoma samples from the ICGC MMML-Seq project [24] were mapped onto the human reference genome hg19 using the splicing-aware mapping tool *segemehl*, version 0.1.7 [20,30] with split-read mapping (option -S) enabled in addition to the default parameters. The total number of reads were around 120 million per sample. The read length was 101 nucleotides, and around 90% of the reads were mappable.

The read supports for all identified splice junctions (i.e., genomic intervals spanning exon–exon boundaries) were calculated. Since *segemehl* identifies split-reads independent of any annotation, we consider all splice junctions located within genomic coordinates taken from GENCODE version 19 as potential introns belonging to that GENCODE gene. In order to call it an intron, we require a minimum of one, five, or ten reads representing the junction. This procedure was performed using the complete range of all dataset sizes from one sample to all 111 samples. Normalized mean expression values—quantified as RPKM (reads per kilobase and million reads)—were averaged over all 111 samples and used to define a gene’s expression level in the lymphoma data set in Figure 2. We utilized the UCSC genome browser [31] to visualize additional introns displayed in Figure 3.

To analyse the annotation landscape of lncRNAs, we used the detailed annotation data from GENCODE (versions 7 through 24) [32], Ensembl (releases 60 and 83) [33,34], NONCODE (version of 2016) [35,36], and the dataset by Cabili et al. [13,37]. We included the categories “antisense”, “lincRNA”, “processed_transcript” and “sense_intronic” biotypes as lncRNAs. The annotation data were used to define the location of individual genes to count the total number of introns. In order to address changes in the annotated gene structures over time, we used the GENCODE v.19 genes as reference. The intersection of GENCODE v.19 with the loci that are supported by at least 10 reads in the lymphoma data set comprises 5257 lincRNAs.

An in-house pipeline was used to aggregate the multiple transcriptomes and compute the summary statistics of interest. Input datasets are provided in the standard Gene Transfer Format (GTF) files, which contain fields indexing the various features of each gene, viz. its exact location, as well as its set of constituent transcripts. Within a gene, transcripts typically overlap and share exons. We therefore first determined a collection of unique exons for each gene, which was subsequently used to determine the unique introns. Mean and median number of introns and exons per gene were computed from the number of unique exons and introns, respectively (i.e., without considering how often they appear in distinct transcripts). A summary of the overlap between lincRNAs of the lymphoma dataset and GENCODE annotations can be found in Table 2.

## Figures and Tables

**Table 1:** Long non-coding RNA (lncRNA) genes catalogued by various annotation systems. The average number of exons and introns per transcript is given in the (avg.) column.

	Genes	Transcripts	Exons	(Avg.)	Introns	(Avg.)
Ensembl 60	1443	1703	4921	2.89	3218	1.88
Cabili 2011	8263	14,353	33,045	2.30	18,607	1.30
NONCODE 2016	160,376	233,696	536,111	2.29	305,771	1.31
GENCODE v7	9580	14,984	42,060	2.81	28,998	1.94
GENCODE v24	15,941	28,031	68,457	2.44	45,016	1.61

**Table 2:** Overlap between lincRNAs expressed in the lymphoma dataset and different versions of the GENCODE annotation.

	Genes	Transcripts	Exons	(Avg.)	Introns	(Avg.)
v7	3296	4563	12,584	2.76	8394	1.84
v19	5257	7487	18,774	2.51	12,010	1.60
v24	4961	7318	18,685	2.55	12,202	1.67

## Abbreviations

The following abbreviations are used in this manuscript:

lncRNA	long non-coding RNA
lincRNA	long intergenic non-coding RNA
rRNA	ribosomal RNA
RPKM	reads per kilobase per million mapped reads
BL	Burkitt lymphoma
FL	follicular lymphoma
DLBCL	diffuse large B cell lymphoma
